# Neuron-NG2 Cell Synapses: Novel Functions for Regulating NG2 Cell Proliferation and Differentiation

**DOI:** 10.1155/2013/402843

**Published:** 2013-08-01

**Authors:** Qian-Kun Yang, Jia-Xiang Xiong, Zhong-Xiang Yao

**Affiliations:** ^1^Department of Physiology, Third Military Medical University, Chongqing 400038, China; ^2^Company 5 of Cadet Brigade, Third Military Medical University, Chongqing 400038, China

## Abstract

NG2 cells are a population of CNS cells that are distinct from neurons, mature oligodendrocytes, astrocytes, and microglia. These cells can be identified by their NG2 proteoglycan expression. NG2 cells have a highly branched morphology, with abundant processes radiating from the cell body, and express a complex set of voltage-gated channels, AMPA/kainate, and GABA receptors. Neurons notably form classical and nonclassical synapses with NG2 cells, which have varied characteristics and functions. Neuron-NG2 cell synapses could fine-tune NG2 cell activities, including the NG2 cell cycle, differentiation, migration, and myelination, and may be a novel potential therapeutic target for NG2 cell-related diseases, such as hypoxia-ischemia injury and periventricular leukomalacia. Furthermore, neuron-NG2 cell synapses may be correlated with the plasticity of CNS in adulthood with the synaptic contacts passing onto their progenies during proliferation, and synaptic contacts decrease rapidly upon NG2 cell differentiation. In this review, we highlight the characteristics of classical and nonclassical neuron-NG2 cell synapses, the potential functions, and the fate of synaptic contacts during proliferation and differentiation, with the emphasis on the regulation of the NG2 cell cycle by neuron-NG2 cell synapses and their potential underlying mechanisms.

## 1. Introduction

Glial cells expressing nerve/glial antigen 2 (NG2 cells) are widespread cell populations identified by their specific expression of NG2 chondroitin sulphate proteoglycan (CSPG), which in the central nervous system (CNS) accounts for approximately 8% to 9% of the total cell population in adult white matter and 2% to 3% of total cells in adult grey matter [1]. These cells mainly differentiate into oligodendrocytes that participate in myelination; their plasticity is manifested by their ability to become astrocytes or neurons under certain conditions [2–4]. NG2 cells have a highly branched morphology, with numerous processes radiating from the cell body [5, 6]. These cells are of particular interest because they exhibit the properties of immature progenitor cells and the physiological features of differentiated mature cells. NG2 cells are considered precursor cells because they can divide, migrate, and finally evolve into myelinating oligodendrocytes [2, 7, 8]. Given that these cells express voltage-gated ion channels, neurotransmitter receptors, and neuron-NG2 cell synaptic contacts, NG2 cells could also be considered to be mature cells [5, 9, 10].

Electrophysiological studies have revealed that NG2 cells express different types of voltage-gated channels in grey and white matter, including the voltage-gated sodium channels (NaV channels) [11], voltage-gated potassium channels [12], and the voltage-dependent calcium channels (VDCC) [13, 14], which are of great significance in regulating the aforementioned cellular activities. 

NG2 cells express ionotropic glutamate receptors (iGluRs) and *γ*-aminobutyric acid (GABA) receptors throughout the CNS [15–17]. Further study indicated that NG2 cells receive functional glutamatergic and GABAergic synaptic inputs from neurons in different brain regions [10, 18–21]. 

Neuron-NG2 cell synapses in the CNS have the following characteristics. (1) Neurons could form classical and nonclassical synaptic junctions with NG2 cells. (2) Neuron-NG2 cell synapses may regulate the NG2 cell cycle in certain ways. During cytokinesis, NG2 cells form cellular processes and synaptic junctions with neurons; some of these synaptic communications, if not all, are eventually passed on to their daughter cells. (3) Neuron-NG2 cell synapses are closely involved in NG2 cell differentiation. Upon differentiation, NG2 cells rapidly lose their functional synapses and develop into mature oligodendrocytes, which participate in the formation of myelin sheaths. 

This review highlights the classical and nonclassical neuron-NG2 cell synapses, the regulatory functions of neuron-NG2 cell synapses on the NG2 cell cycle, and the fate of synaptic junctions during NG2 cell proliferation and differentiation, with an emphasis on the potential functions of neuron-NG2 cell synapses for regulating the proliferation and differentiation of NG2 cells.

## 2. Neuron-NG2 Cell Synapses in CNS

### 2.1. Classical and Nonclassical Neuron-NG2 Cell Synapses in CNS

 Neuron-NG2 cell synapses are ubiquitously found throughout the CNS. Based on traditional neuron-neuron synapse characteristics, neuron-NG2 cell synapses can be briefly classified into two types: classical and nonclassical. The former shares the features of the traditional neuron-neuron synapse, both in terms of its morphology and physiology. The latter differs in its anatomical structures and physiological functions. 

**Figure 1:**
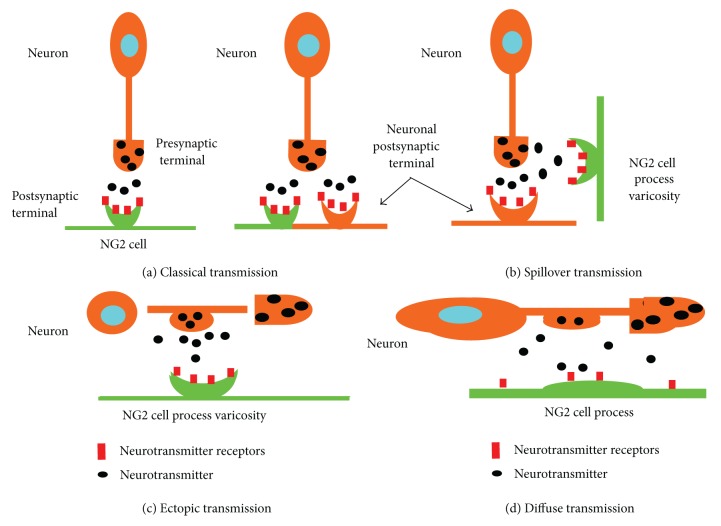
Potential modes of classical and nonclassical neuron-NG2 cell synapse transmissions. (a) Classical transmission. The neuron axon or cell body forms close contacts with NG2 cell processes or cell bodies, thereby forming specialized synaptic junctions similar to neuron-neuron synapses. A presynaptic terminal can simultaneously innervate two or more NG2 cells, or an NG2 cell process and a neuron spine (right). Neurotransmitters released from the neuronal terminal can reach the receptors of postsynaptic structures, leading to a cascade of NG2 cell activities. (b) Spillover transmission. Variations on the NG2 cell processes, located beside the neuron-neuron synapse, form an enigmatic communication structure. The neurotransmitters from the synaptic cleft diffuse outside and activate the receptors on the varicosities of NG2 cells. (c) Ectopic transmission. The variations of neuron axons and NG2 cell processes form close contacts, thereby leading to ectopic transmission. The neurotransmitters mainly come from the synaptic-like vesicles of neuron axon varicosities. (d) Diffuse transmission. Receptors on NG2 cell processes are divergent, and the neurotransmitters may have multiple sources. Neurotransmitters must diffuse from a large distance before reaching a target cell; thus, the reaction may be not as strong as that in other transmission modes.

Classical synaptic transmission between neurons and NG2 cells is similar to the traditional neuronal synapses. These shared characteristics include the rigid alignment of neuron and NG2 cell membranes, the existence of an active zone with characteristic synaptic vesicles on the neuronal side, the space occupied by neuron-NG2 cell synapses, and the dense postsynaptic density (PSD) on the side of the NG2 cells [22–24]. Axons with vesicle-containing presynaptic compartments directly form contacts with NG2 cell processes to form specialized synaptic junctions; the released neurotransmitters can diffuse across the narrow cleft to directly activate high densities of postsynaptic receptors in NG2 cells [24, 25]. A single presynaptic button can simultaneously innervate a neuronal spine and the individual or multiple NG2 cell process (Figure 1(a)) [26–28]. Consistent with these data, previous evidence has suggested that the glutamate alpha-amino-3-hydroxy-5-methyl-4-isoxazole propionic acid (AMPA) receptors are not uniformly expressed over the surface of NG2 cells; these structures are instead clustered into discrete plaques along the processes [24, 25, 29]. Freeze-fracture immunolabeling of brain tissue from transgenic mice allows the clear identification of NG2 cells using anti-GFP antibodies; the results showed clusters of AMPA receptor-immunoreactive particles along the NG2 cell processes [30]. What is more, these AMPA receptors show different levels of Ca^2+^ permeability in different stages of development [31].

These findings indicate that NG2 cells are morphologically direct targets of innervations. Furthermore, AMPA receptor signaling happened at discrete locations where NG2 cells and axons formed bona fide synaptic junctions, rather than diffusely occurring over the surface of NG2 cells.

Patch-clamp recordings of NG2 cells from the hippocampus have demonstrated the existence of spontaneous rapid AMPA and GABA receptor-mediated currents [17, 22, 23]. These currents in grey matter regions exhibit the typical characteristics of classical neuron-neuron synaptic events. Specifically, they occur with minimal delay after the action potentials are triggered in the surrounding axons and can be stably evoked by a single action potential [18, 19]. The spontaneous events induced by the unsynchronized release of neurotransmitters from the presynaptic contacts are visible when action potentials are inhibited using tetrodotoxin (TTX); these events are clearly facilitated in response to repetitive stimulation by amplitude fluctuations [18]. Taken together, these data suggest that NG2 cells indeed form classical synaptic contacts with neurons, particularly with neuronal axons. 

Compared with classical synapses, the more common forms of synaptic contact between neurons and NG2 cells are the nonclassical neuron-NG2 cell synapses, such as the ectopic, spillover, and diffuse transmissions.

Neurotransmitters could be found in small “synaptic-like” vesicles, which are located at varicosities along axons and released at extrasynaptic sites [24]. High densities of extrasynaptic receptors can be activated adjacent to these release sites to induce rapidly accumulating discrete events in NG2 cells. However, these events have apparently smaller amplitudes, as compared with those recorded at classical neuron-NG2 cell synapses [25]. This form of signal communication is referred to as ectopic transmission (Figure 1(c)). The quantal release of glutamate from axons to NG2 cells in the white matter region has been recently reported during postnatal development [21, 25]. This discovery indicated that white matter in the brain is where bundles of axons connect different brain regions and where local information may be accurately transferred and integrated at specialized sites between neurons and NG2 cells. The white matter region in the corpus callosum is responsible for interhemispheric communication; the glutamate released by vesicular fusion in callosal axons elicits quantal AMPA receptor-mediated currents in NG2 cells at anatomically distinct axon-NG2 cell synaptic contacts, which is a typical form of ectopic transmission [24, 25]. 

Another mode of signal transmission between neurons and NG2 cells is spillover transmission (Figure 1(b)). NG2 cell processes are found close to neuron-neuron synapses, thereby allowing high density of extrasynaptic receptors to sense local neurotransmitters spilling out of the synaptic cleft [26]. Spillover transmission may be correlated with neurotransmitter dynamics and neuronal information processing. This kind of synaptic transmission has been reported in NG2 cells of the barrel cortex of NG2-DsRed transgenic mice [32].

Compared with other synaptic transmissions, diffuse transmission may occur at nonsynaptic or extrasynaptic sites; the neurotransmitters must diffuse across relatively longer distances before reaching a target cell, which has divergent neurotransmitter receptors (Figure 1(d)) [26, 33, 34]. NG2 cells have been reported to express AMPA and GABA receptors, but these cells also express other functional receptors on their membranes, such as the *N*-methyl-D-aspartate receptors (NMDARs) [35, 36], the kainate receptors [37] of glutamate, the ionotropic nicotinic acetylcholine receptors (nAChRs), and the purinergic receptors, which also play a vital part in information transmission from neurons to NG2 cells [38, 39]. Considering the highly branched morphology of NG2 cells, it is plausible that this kind of synaptic transmission may be a more universal phenomenon in CNS. 

### 2.2. General Functions of Neuron-NG2 Cell Synapses

 Although the existence of neuron-NG2 cell synapse is a universal phenomenon, the functions of the said synapse remain unknown. Elucidating this question for the complexities and diversities of neuron-NG2 cell synapses is rather difficult. However, previous studies have laid the basic foundations for this problem. Neuron-NG2 cell synapses may have the following potential functions. First, the synaptic inputs from neurons in physiological and pathological conditions may directly regulate the activities of NG2 cells, such as cell proliferation, migration, and differentiation [15, 21, 40–43]. Second, neuron-NG2 cell synapses may be involved in the transformation or integration of neuronal signals propagating along axons at specialized sites on the neuron-NG2 cell synaptic contacts [24, 25]. Third, myelination may be initiated in sites where synaptic junctions are formed, given the diversity of synaptic contacts between neurons and NG2 cells, particularly the nonclassical synapses. NG2 cells in CNS are influenced by neuronal activities; thus, they can rapidly react to changes within the CNS. This interaction between neurons and NG2 cells is highly efficient in physiological and pathological conditions. For example, demyelinated neurons could regulate NG2 cell activities through a variety of ways, such as differentiation into mature oligodendrocytes and myelin sheath formation [44–46]. Consequently, NG2 cells are more efficient than neural stem cells from the subventricular zone in terms of differentiating into oligodendrocyte precursor cells, migrating to targeted axons, and participating in myelination. 

A series of studies have indicated that NG2 cell activities are closely related to neuron-NG2 cell synapses. However, the mechanisms by which neurons regulate the actions of NG2 cells remain to be elucidated. To date, several studies have laid the groundwork for this problem. We will discuss these findings in detail in this review, especially concentrating on the detailed mechanism of NG2 cell cycle regulation. 

### 2.3. Neuron-NG2 Cell Synapses Modulate NG2 Cell Cycle and Proliferation

#### 2.3.1. NG2 Cell Cycle

**Figure 2:**
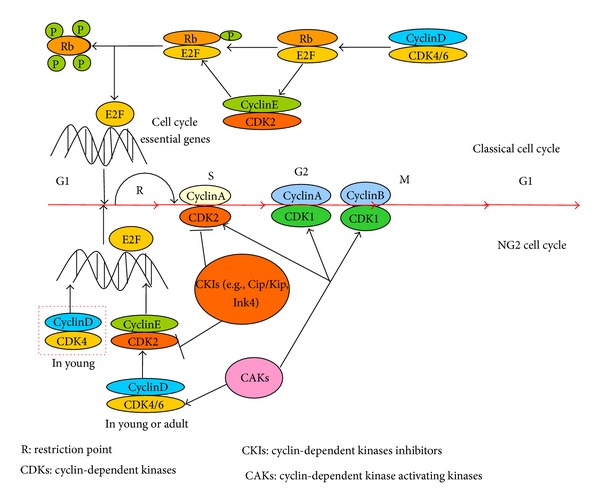
Regulation of the NG2 cell cycle. The main difference between the NG2 cell cycle (lower part) and classical cell cycle (upper part) lies in the fact that CDK2 is not essential in the earlier stage of the NG2 cell cycle because of the compensatory function of CDK4 (red dotted line box). Aside from the said observation, there are no further differences reported between the NG2 cell cycle and the classical cell cycle. Both the NG2 cell cycle and the classical cell cycle are regulated by cyclin-dependent kinase activating kinases (CAKs) and CDK-inhibitors (CKIs). The former mainly promote the progression of the NG2 cell cycle, whereas CKIs inhibit cell division or induce cell differentiation. The extracellular signals mainly act on CKIs or CAKs, thereby altering the fate of NG2 cells.

The classical cell cycle in rodents is driven by the coordinated activities of cyclin-dependent kinases (CDKs) and their cyclin substrates [47]. During the G_1_ phase, the CDK4/6-cyclin D complexes gradually phosphorylate the retinoblastoma protein (Rb), causing E2F transcription factors to promote the expression of essential cell cycle genes such as cyclin E. Cyclin E binds with CDK2 to form the CDK2-cyclin E complexes, which then phosphorylate Rb and consequently completely inactivate of Rb as well as the subsequent expression of proteins necessary for DNA replication in the S phase [48, 49]. CDK2 and cyclin A both promote the progress of cells during the S phase [50]. Cyclin A then binds with CDK1 to form CDK1-cyclin A complexes at the end of the S phase. The CDK1-cyclin A complexes then facilitate the progression to G_2_ phase. Finally, CDK1-cyclin B complexes induce the G_2_-M transition as well as modulate the structural and regulatory events during mitosis (Figure 2, upper portion). CDK activity is precisely regulated by CDK activating kinases (CAKs) and CDK inhibitors (CKIs), such as Ink4 and Cip/Kip family [51–54]. CKIs inhibit cell cycle progression, inducing cells to exit the cell cycle and remain as quiescent cells or to undergo differentiation. These elements contribute towards fine-tuning the advancement through the different cell cycle phases, which finally leads to mitotic cell division. However, whether the NG2 cell cycle follows a similar mechanism has yet to be determined.

The NG2 cell cycle is regulated by the successive expression of CDKs and cyclins; a series of studies highlight the functions of CDK2. Compared with the classical cell cycle, regulation of the NG2 cell cycle has unique characteristics (Figure 2, lower part). Studies revealed that CDK2 is essential for the proliferation and self-renewal of NG2 cells in adults, whereas it is dispensable for the early generation of oligodendrocytes because of the compensatory mechanisms of CDK4 activation [55–57]. Developmental studies have shown that CDK2^−/−^ and wild-type mice at perinatal ages have no significant differences in the density and number of proliferative NG2 cells in the subventricular zone (SVZ), whereas the proliferation indices are significantly reduced in adult CDK2^−/−^ mice [58]. Further study using cultured cells of adult CDK2^−/−^ SVZ indicated that NG2 cells demonstrate a decreased capacity for self-renewal but show enhanced differentiation. The compensatory mechanisms of CDK4 activation persist until postnatal day 15, and a decrease of CDK4 expression was clearly observed in postnatal day 28, as the NG2^+^ proliferation and self-renewal of CDK2^−/−^ cells were inhibited [56]. Consistent with this data, CDK4 silencing of perinatal CDK2^−/−^ SVZ cells largely reduced cell proliferation and self-renewal in adults, whereas CDK4 overexpression renewed the proliferative capacity to wild-type levels [55]. Therefore, CDK2 is less important for the NG2 cell cycle transition from the G_1_ to S phase in the early stage, whereas the said factor is critical for adult progenitor cell proliferation and self-renewal. The differences in CDK2 expression and activity may underlie the NG2 cell activities in vivo.

#### 2.3.2. Neuron-NG2 Cell Synapses Regulate NG2 Cell Cycle and Proliferation

**Figure 3:**
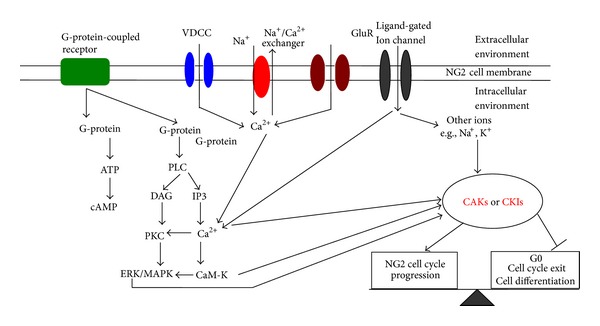
Possible signaling pathways of synaptic transmitters to activate the progression of NG2 cell cycle. Extracellular signals through different pathways, such as VDCCs, activation of GluRs, ligand-gated ion channels, and G-protein-coupled receptors, can finally affect the activities of CAKs or CKIs. CAKs or CKIs then act on CDKs or cyclins to determine NG2 cell cycle progression or exit. Other pathways are not excluded. AC: adenylate cyclase; CAKs: cyclin-dependent kinase activating kinases; CaM-K: calmodulin kinase; CKIs: cyclin-dependent kinases inhibitors; DAG: diacylglycerol; ERK/MAPK: extracellular signal-regulated kinase/mitogen-activated protein kinase; PKC: protein kinase C; PLC: phospholipase C.

During the development of NG2 cells, the cell cycle is precisely regulated by a complex array of signaling systems, including electrical and chemical signals from the surrounding neurons or glia [13]. The electrical signals mainly arise from the VDCCs [13, 14], sodium channels [5, 59], complex family members of potassium channels, and ligand-gated channels [60, 61]. These chemical signals mainly consist of growth factors and neurotransmitters (Figure 3). 

Calcium signaling has been reported to be extensively involved in oligodendroglial lineages, such as in cell cycle regulation as well as cell proliferation, migration, and differentiation. Calcium can enter NG2 cells through multiple pathways, including ligand-gated channels, such as the glutamate receptors [26], P2X and P2Y purinoceptors with ATP as ligand [62–64], *α*-adrenergic receptors [65, 66], VDCC, or released from endoplasmic reticulum (ER). [Ca^2+^]_i_ transients are required for the proliferative cell cycle to progress, including the G_1_/S-phase transition, S-phase, and mitosis, as well as key points within mitosis such as the metaphase-anaphase transition and the induction of cytokinesis. Neurotransmitters such as glutamate are released from presynaptic terminals and may activate glutamate receptors on NG2 cells, thereby causing an influx of Ca^2+^. Other neurotransmitters (e.g., GABA) or growth factors including the platelet-derived growth factor (PDGF), basic fibroblast growth factor (bFGF), ciliary neurotrophic factor (CNTF), and neurotrophin-3 (NT-3) are released from neighboring axons and can induce the Ca^2+^ influx through various mechanisms. 

Neurotransmitter-mediated [Ca^2+^]_i_ increase initiates a sequence of downstream consequences, from gene activation to cell cycle progression, which depends on the precise timing of these signals. Studies have shown that the activation of GluR causes a large influx of Na^+^ and Ca^2+^ ions through the cell membrane by blocking K^+^ currents in NG2 cells, thereby leading to the depolarization and inhibition of cell proliferation [67, 68]. The depolarization of NG2 cells may be accomplished by the increasing expression of CKIs expression, such as p27 and p21, which then causes cells to exit the cell cycle or differentiate [69]. Further studies revealed that glutamate AMPA receptor-mediated calcium signaling is transiently enhanced during the development of oligodendrocytes. This observation is not due to the decrease in GluR2 expression because the real-time PCR analysis of different NG2 cell developmental stages suggested that the expression AMPA-GluR2 subunit remains relatively constant. However, GluR3 and GluR4 expressions are significantly increased in oligodendroglial progenitors and immature oligodendrocytes [43, 70], which may account for the increased susceptibility of NG2 cells to excitotoxicity, as compared with mature oligodendrocytes.

Neurotransmitters can bind with G-protein-coupled receptors and lead to the conversion of ATP to cAMP; alternatively, neurotransmitter binding triggers the phosphorylation of phospholipase C (PLC), activation of protein kinase C (PKC), and stimulation of the extracellular signal-regulated kinase (ERK)/mitogen-activated protein kinase (MAPK) pathway in NG2 cells [71]. For example, stimulation of the P2Y receptor activates the IP3/Ca^2+^ signaling cascade [39] and the subsequent initiation of the ERK/MAPK pathway. The ERK/MAPK pathway is closely associated with cell cycle progression because it primarily suppresses the cell cycle blockades at the step between the G_1_ and S phase [72]. Thus, the ATP-induced cell proliferation in NG2 cells is likely mediated via the PKC/MAPK pathway. The same signaling cascade has been reported to facilitate the ATP-mediated cell proliferation of astrocytes [73–75]. A similar MAPK-dependent signaling mechanism has been associated with the control of the proliferation in astrocytes and other cell lines by acetylcholine and glutamate [76, 77]. 

 Except for glutamate receptors, the VDCCs are important in regulating calcium levels in the CNS and have vital functions in NG2 cell proliferation. Six types of VDCCs (namely, P/Q, N, L, R, and T) have been classified based on their electrophysiological and pharmacological properties [14]. Immunohistochemical studies have reported the expression of L-, N-, and R-type VDCCs in oligodendrocyte lineages in vivo [63]. Subsequent studies reported that NG2 cell depolarization could lead to the opening of VDCCs, thereby causing an influx of Ca^2+^. Neurotransmitters such as glutamate, ATP, and growth factors could modulate this process in various ways. The increased Ca^2+^ concentration then functions as a secondary messenger to activate downstream signaling pathways that eventually fine-tunes the cell cycle. 

The phosphatidylinositol 3-kinase (PI3K)/Akt pathway is activated by extracellular mitogens and involved in various cell activities. This pathway regulates multiple cellular processes including cell proliferation, survival, and growth. Downstream targets of PI3K include the protein kinases, such as Akt and S6 kinases, as well as small GTPase regulators [78, 79]. ATP may act via the metabotropic P2Y purinoceptors to promote the proliferation of neural stem cells via the PI3K-dependent pathway [80, 81]. The PI3K pathway is likewise involved in mediating mitogenic signals after P2Y receptor activation in the Müller cells [82, 83]. Thus, the PI3K-dependent pathway may also regulate the NG2 cell cycle in a similar manner. 

The regulatory functions of neuron-NG2 cell synapses on the NG2 cell cycle are of great significance in CNS. During brain development, neurons derived from neural stem cell niches project their axons to targeted areas. NG2 cells may migrate along these axons via mechanical traction or signals in the axon-NG2 cell synapse. NG2 cells can differentiate into oligodendrocytes and consequently enwrap axons participating in myelination. Mangin et al. [84] recently found that sensory inputs from thalamocortical fibers regulate the proliferation and localization of NG2 cells in the mouse somatosensory cortex barrel field. Electrophysiology analysis showed that thalamocortical afferents originate from the ventrobasal part of the thalamus, convey inputs from the whisker fields, and form synapses to NG2 cells. Astrocytes accumulate in the center of the barrels, whereas NG2 cells are enriched in the border regions of the barrel walls and the septa when the barrel is formed between postnatal days 4 and 6. When a row of whiskers or the entire whisker pad was cauterized, thalamocortical input into the corresponding barrels was reduced. The reduced synaptic inputs were found to increase the proliferation rates of NG2 cells at the center of the barrels, which sequentially produced a more uniform distribution of NG2 cells in the activity-deprived barrels. These data suggested that synaptic signals are closely correlated with NG2 cell activities. Furthermore, the continued presence of NG2 cells as oligodendrocyte progenitors in the adult brain may be a prerequisite for the continued, although reduced, plasticity of some brain regions such as the adult cerebral cortex. Thus, NG2 cells may be involved in learning and memory by improving neural circuits via the modulation of myelination and, consequently, the conduction velocity and/or timing. 

### 2.4. NG2 Cells Maintain Synaptic Contacts during Mitosis and Pass Them to the Resulting Daughter Cells

NG2 cells are progenitor cells capable of self-renewal. The proportion of actively cycling NG2 cells is relatively constant and high, both in young (~1 to 2 weeks old) and in adult (~18 months old) rodents [21, 85]. Double staining for the NG2 protein and the proliferating cell nuclear antigen (PCNA) was performed to investigate the fraction of actively cycling cells in the brain. PCNA can only be detected in proliferative cells. Kukley et al. [21] observed that the fraction of NG2 cells in cell cycle (NG2+ and PCNA+) at P9 and P11 in the mouse hippocampus reaches as high as 48% and 49%, respectively. A subsequent study reported that the proportion of actively cycling cells at P6 in the murine corpus callosum is approximately 55% [85]. These data indicated that NG2 cells are actively proliferating in the CNS. 

During mitosis, NG2 cells maintain their morphological and physiological characteristics, including the highly branched cellular processes and voltage-gated ion channels as well as the active glutamatergic and GABAergic synaptic inputs. Morphological analysis of NG2 cells stained with the fluorescent dye Lucifer yellow revealed that NG2 cells have a rich tree of branching processes during metaphase and telophase [21]. Moreover, electron microscopy revealed that synaptic terminals come into contact in mitotic NG2 cells, thereby demonstrating that certain, if not all, synaptic contacts are maintained during cell division [86]. Patch-clamp recordings have been performed using mitotic NG2 cells (metaphase or telophase of mitosis) from acute hippocampal and cortical slices of neonatal (7 to 12 days old) mice. The AMPA/kainate receptor antagonist 6-cyano-7-nitroquinoxaline-2,3-dione (CNQX) allows for the GABAergic synaptic current that was clearly detected in NG2 cells with mitotic chromosome configurations. When the AMPA/kainate receptor antagonists are removed from the extracellular solution and the GABA receptor antagonist bicuculline is subsequently added, the rapid rise and subsequent decay of excitatory postsynaptic currents were observed in NG2 cells during the mitotic metaphase and telophase [21, 87]. Similar findings were reported by Ge et al. [86] who demonstrated that NG2 cells were undergoing mitotic metaphase and telophase in the grey and white matter in the brain. These cells receive glutamatergic synaptic inputs from neurons in young and older animals (up to 20 weeks old). 

In addition, certain observations also provide indirect evidence for the inheritance of synaptic contacts during mitosis. First, the frequency of synaptic current is comparable between the metaphase and telophase cells [21, 86]. Two daughter cells in telophase are connected by a large cytoplasmic bridge. Thus, the recorded current is hypothesized to be from both progeny cells. Second, morphological analysis indicated that each daughter cell has approximately half the number of glutamic acid decarboxylase 65- (GAD65-) positive putative GABAergic terminals in the parent cell [21, 88]. Altogether, these data indicated that NG2 cells are continuously kept functional; certain synaptic contacts are passed onto the resulting progenies during cell division. 

**Figure 4:**
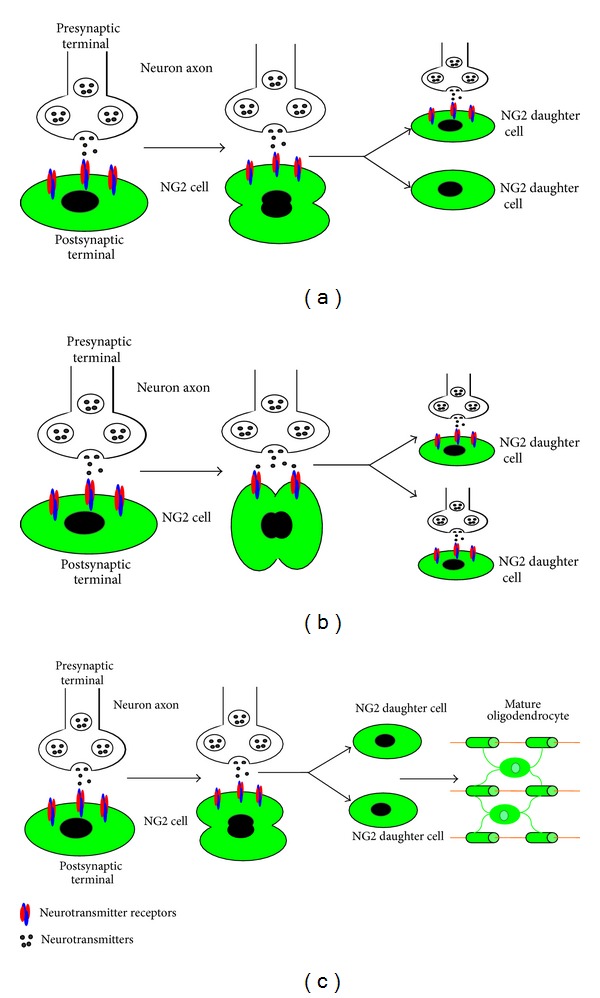
Schematic diagram of NG2 cell division. (a) An NG2 cell divides, with one of the resulting daughter cells keeping the functional synapse and the other loses synaptic contact with the neuron. (b) An NG2 cell maintains synaptic contacts and passes these onto the two progeny cells. (c) NG2 cells lose synaptic input from neurons and differentiate into mature oligodendrocytes, enwrapping axons to form myelin sheaths.

Experiments have found that NG2 cells inherit synaptic contacts from the respective parent cells, but the same is not true for all proliferative NG2 cells. There are three possibilities during the process of NG2 cell division (Figure 4). First, an NG2 cell divides into two daughter cells and one of them keeps the functional synapse while the other loses synaptic contact with the neuron. The latter may further differentiate into mature oligodendrocyte or undergo apoptosis (Figure 4(a)). Second, both daughter cells inherit the synaptic contacts from their parent cell. These cells may continue to function as progenitor cells for future activities including the compensation for myelination through differentiation under physiological and pathological conditions (Figure 4(b)). Third, both daughter cells lose synaptic contacts with neurons and differentiate into myelinating oligodendrocytes, thereby participating in the myelination of axons (Figure 4(c)). 

Synaptic transmission between neurons and NG2 cells raises question of the significance of the said signaling input on NG2 cells. Keeping synaptic contacts with dividing NG2 cells may give neurons the opportunity to directly or indirectly regulate the activities of NG2 cells. For example, the synaptic signal from neurons may influence the potassium channels of NG2 cells during the cell cycle and/or via the control of other intracellular signal cascades [89]. Previous studies have shown that voltage-gated potassium channels (Kv1.3) are important for controlling the complement C5b-9-induced cell cycle activation, both in vitro and in vivo [90]. The complement C5b-9 can induce the increased expression of Kv1.3 channels and the subsequent activation of the ERK1 and PI3K/Akt pathways, thereby contributing to the survival of NG2 cells. Meanwhile, the inhibition of Kv1.3 channels causes an accumulation of the CKIs, such as p27 and p21, which then leads to G_1_ arrest in NG2 cells [68, 91, 92]. Furthermore, the synaptic signals from neurons may determine the fate of NG2 cells, such as self-renewal, differentiation, and even transformation to oligodendrogliomas. The proteoglycan present on the NG2 cell membrane, together with the glutamate receptor interacting protein (GRIP), forms the NG2-GRIP-AMPA receptor complexes, which are involved in the alignment and formation of the neuron-NG2 cell synapse [30]. The expression of these receptors is downregulated as the NG2 cells mature into oligodendrocytes. Previous studies have shown that oligodendrocyte differentiation from adult multipotent stem cells is modulated by glutamate [93]. Therefore, the disruption or the internalization of NG2-GRIP-AMPA receptor complexes may be associated with the differentiation of NG2 cells. Furthermore, research also has shown that the NG2 proteoglycan segregate asymmetrically during mitosis [94]. The NG2 protein is required for asymmetric segregation of the epidermal growth factor receptor (EGFR). EGFR influences cell fate choices in embryonic glial progenitor cells and adult neural stem cells; it is also essential for normal tissue functions. EGFR enhances adult oligodendrogenesis and myelination, whereas constitutive EGFR signaling in NG2 cells prevents their differentiation, which may leads to hyperplasia. The decreased NG2 asymmetry coincides with the premalignant abnormal self-renewal instead of differentiation and demonstrates tumor-initiating potential. Thus, the loss of synaptic contacts may cause the dysfunction of the NG2 protein, thereby leading to transformation from normal NG2 cells to the cellular origin of the neoplasm. Modulation of NG2 proteoglycan may be a novel potential target for the treatment of NG2 cell-related tumors. 

## 3. Neuron-NG2 Cell Synapses Are Rapidly Decreased during Cell Differentiation

**Figure 5:**
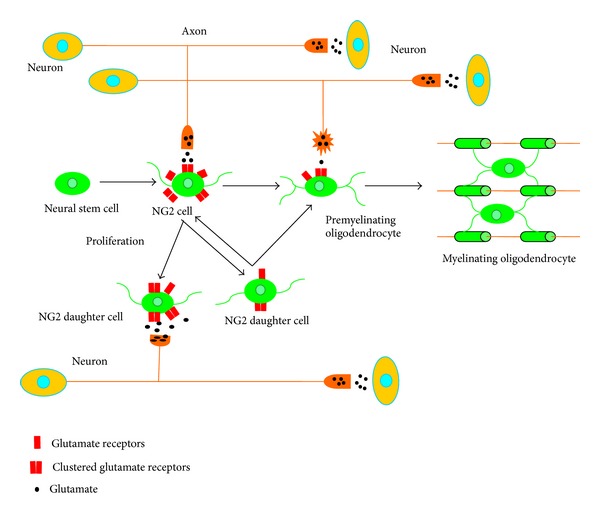
Fate of neuron-NG2 cell synapses during NG2 cell proliferation and differentiation. NG2 cells keep functional synapses during division; NG2 daughter cells could also form synapses with neurons or undergo differentiation. When NG2 cells differentiate into premyelinating oligodendrocytes, the synaptic contacts between neurons and NG2 cells were rapidly decreased, accompanied with the disruption of synaptic structures and the decrease of glutamate receptors on NG2 cells. As premyelinating oligodendrocytes grow into mature oligodendrocytes, the processes of oligodendrocytes contact neuronal axons and form myelin sheaths. This process may be regulated by other modes of signal transmission as well.

NG2 cells have the potential to differentiate into mature oilgodendrocytes, which then come into contact with axons and subsequently enwrap them to form the myelin sheath. Before the formation of a functional myelin sheath, NG2 cells passes through two developmental stages, namely, premyelinating oilgodendrocytes and mature oligodendrocytes [8, 95, 96]. During the process of NG2 cell differentiation, the synaptic communication between neurons and NG2 cells is rapidly lost (Figure 5) [87, 88]. The differentiation of NG2 cells is characterized by the expression of stage-specific proteins, which are finely tuned by a series of transcription factors and accompanied by the expression of specific molecular markers [8, 97–99]. Morphological studies demonstrated that premyelinating oligodendrocytes are not found in NG2 and PDGFR*α*. These cells do not display myelin sheaths but are positively stained by the O1 antibodies and proteolipid protein (PLP)/DM20 antibodies [87]. PLP is a highly conserved tetraspan protein that functions as the major structural component of myelin in the CNS; DM20 is a smaller splice isoform of PLP [100]. Mature myelinating oligodendrocytes possess fewer processes than NG2 cells or premyelinating cells, but they show numerous myelin sheaths [87, 88].

Subsequent electrophysiological studies likewise provide evidence for the decreased number of neuron-NG2 cell synapses during NG2 cell differentiation. Two different methods were employed to probe vesicular neurotransmitter release in neuron-glia synapses at distinct stages of differentiation in different brain regions in situ. Electrophysiological analysis indicated that the spontaneous synaptic responses in all NG2 cells can be detected, whereas only a few premyelinating cells displayed synaptic currents. None of the currents could be induced in mature myelinating oligodendrocytes [101]. Similar results were observed in another study, wherein extracellular stimulation to synchronously induced action potentials in a large number of surrounding axons to test the synaptic input onto an identified NG2 cell [22, 87]. When axons were stimulated electrically while recording an NG2 cell, the obvious synchronous compound response to neurotransmitters released from hundreds of nearby synaptic terminals could be observed in the NG2 cell. Synaptic response in the NG2 cell has a brief latency (<5 ms) following electrical stimulation, instantly increasing to a sharp peak (<1 ms rise time from 20% to 80%) and then rapidly decayed back to the baseline (decay time constant is approximately from 2 to 4 ms). Compared with NG2 cells, it was not possible to elicit any synaptic responses in the mature myelinating oligodendrocytes and in majority of the premyelinating oligodendrocytes [87]. Altogether, these data demonstrated that synaptic signals between neurons and oligodendrocyte lineage cells are rapidly decreased during NG2 cell differentiation. 

Several questions remain. Does the loss of functional synapses in NG2 cells result from the decrease in neurotransmitters present or the changes in receptor subunits on the cell surface? Or may the changes in the cell voltage-gated sodium, potassium channels, the disruption of the synapse structures, or the changes of synaptic transmission modes result in the observed loss? Hitherto, the mechanism underlying the rapid decrease of synaptic signaling and the significance of these changes remain unknown. But there may be some possible explanation for the phenomenon. 

Application of glutamate via the fast photolysis was conducted to probe the aforementioned questions. NG2 cells and premyelinating oligodendrocytes display clear currents, which are prevented by the AMPA/kainate receptor antagonists. However, the amplitude of these currents is much smaller in the premyelinating cells than in the NG2 cells [87, 101]. The amplitude of AMPA/kainate receptor-mediated current decreases as premyelinating cells differentiate into mature cells and oligodendrocytes [87]. Coincidentally, the differentiation of NG2 cells is accomplished by a decrease in the NG2 proteoglycan, which is important in the dynamic regulation of AMPA receptors [102]. Thus, the downregulation of NG2 proteoglycan may result in the internalization or disruption of AMPA receptors,which may lead to the decrease of neuron-NG2 cell synapses. 

Furthermore, developmental studies demonstrated that the loss of these synaptic signals coincides with a downregulation of voltage-gated sodium and potassium channels. Apparent loss of Na^+^ currents in NG2 cells was observed during the process of differentiation. Voltage-gated Na^+^ channel-as well as mediated currents blocked by TTX is examined in P3–P8 mice, whereas Na^+^ currents are apparently decreased in cells, as recorded in P10–P18 mice [9, 11, 103]. These results illustrate that sodium channel expression is downregulated in situ; thus, the currents presented in NG2 cells disappear during the development of the oligodendrocyte lineages.

The signal transmission mode between neurons and premyelinating oligodendrocytes possibly shifts from synaptic to extrasynaptic transmission, such as ectopic, spillover, or diffuse transmission. The NG2 cells of the barrel cortex of NG2-DsRed transgenic mice have been reported to receive GABAergic synaptic inputs from interneurons [32]. During development, the frequency of spontaneous and low GABAergic synaptic activity in cortical NG2 cells was dramatically decreased after the second postnatal week. However, NG2 cells still receive GABAergic inputs from interneurons in the adult cortex. These inputs do not rely on the presence of functional synapses but depend on the form of GABA spillover. The GABA spillover allows interneurons to elicit responses in target NG2 cells by the activation of extrasynaptic GABA receptors. Thus, there is a possible change in the synaptic transmission mode during CNS development. 

Although previous studies could explain some of the observed phenomena, there is no definite explanation for these questions. Systematic and developmental studies will help unravel these enigmatic problems. 

## 4. Conclusions and Perspectives

The formation of neuron-NG2 cell synapses is a universal phenomenon in CNS. NG2 cells could form classical and nonclassical synaptic contacts with neurons throughout the brain, regardless of their age. A series of studies have revealed that synaptic inputs from neurons may directly regulate NG2 cell activities, such as cell proliferation, migration, and differentiation. However, the detailed mechanisms involved remain largely unknown. Given the complexity and diversity of neuron-NG2 cell synapses, these synaptic contacts may have greater significance, as compared with other synapses. First, the nonclassical synaptic contacts are more universal in CNS, but their significance remains unknown. Almost all NG2 cells may be correlated with neurons, which fine-tune the NG2 cell activities. Second, NG2 cells have highly branched processes and mainly form synapses with axons in the white matter. The nonclassical synapses are coincidentally found to mainly exist along the NG2 cell processes. The various neuronal axons release neurotransmitters such as glutamate to bind with the receptors on the surface of NG2 cell membrane, thereby influencing NG2 cell activity. Thus, oligodendrocytes probably initiate myelination at these sites while the mature oligodendrocytes continue to communicate with neurons via nonclassical synaptic transmission. Third, neuron-NG2 cell synapses are involved in NG2 cell cycle regulation. However, the details of the regulatory signaling pathways have not been elucidated to date. Future studies on NG2 cell cycle regulation will provide a foundation for the treatment of diseases caused by CNS demyelination, such as hypoxia-ischemia injury, periventricular leukomalacia, and multiple sclerosis, among others. NG2 cells are severely damaged by hypoxia-ischemia injury and periventricular leukomalacia. Thus, the remaining NG2 cells are not enough to function as oligodendrocyte precursor cells for myelination and remyelination. By contrast, the fate choices of NG2 cells may have been altered in multiple sclerosis, such that they are unable to perform their physiological functions in the CNS. These diseases are closely related to NG2 proliferation and differentiation. Regulation of the NG2 cell cycle may be a potential therapeutic target to address these problems. Moreover, NG2 cells have been correlated with plasticity in certain brain regions such as the adult cerebral cortex. Synaptic inputs onto NG2 cells may improve the efficiency of neural circuits by modulating myelination and, in turn, the conduction velocity. Therefore, understanding the link between neurons and NG2 cells is important for elucidating the regulatory mechanisms of the NG2 cell cycle and improving the prognosis of demyelinated diseases, as well as explaining the molecular foundations of learning and memory. 

## Abbreviations

AMPARs: Alpha-amino-3-hydroxy-5-methyl-4-isoxazole propionic acid receptors
bFGF: Basic fibroblast growth factor
CAKs: CDK activating kinases
CDKs: Cyclin-dependent kinases
CKIs: CDK inhibitors
CNQX: 6-Cyano-7-nitroquinoxaline-2,3-dione
CNS: Central nervous system
CNTF: Ciliary neurotrophic factor
CSPG: Chondroitin sulphate proteoglycan
DsRed: Red fluorescent protein
EGF: Epidermal growth factor
EGFR: Epidermal growth factor receptor
ER: Endoplasmic reticulum
ERK/MAPK: Extracellular signal-regulated kinase/mitogen-activated protein kinase
GABA: *γ*-Aminobutyric acid
GAD65: Glutamic acid decarboxylase 65
GRIP: Glutamate receptor interacting protein
iGluRs: Ionotropic glutamate receptors
nAChRs: Nicotinic acetylcholine receptors
NaV channels: Voltage-gated sodium channels
NG2: Nerve/glial antigen 2
NMDARs: N-methyl-d-aspartate receptors
NT-3: Neurotrophin-3
PCNA: Proliferating cell nuclear antigen
PDGF: Platelet-derived growth factor
PI3K: Phosphatidylinositol 3-kinase
PKC: Protein kinase C
PKC/MAPK: Protein kinase C/mitogen-activated protein kinase
PLC: Phospholipase C
PLP: Proteolipid protein
PSD: Postsynaptic density
SVZ: Subventricular zone
TTX: Tetrodotoxin
VDCC: Voltage-dependent calcium channels.
